# Parent artery occlusion for ruptured aneurysms in moyamoya vessels or on collaterals

**DOI:** 10.3389/fneur.2023.1085120

**Published:** 2023-01-30

**Authors:** Zibo Zhou, Kan Xu, Jinlu Yu

**Affiliations:** Department of Neurosurgery, The First Hospital of Jilin University, Changchun, China

**Keywords:** moyamoya disease, aneurysm, collateral, parent artery occlusion, outcome

## Abstract

**Background:**

Aneurysms in moyamoya vessels or on collaterals are difficult to treat. Parent artery occlusion (PAO) *via* endovascular treatment (EVT) is often the last resort, but the safety and efficacy of this approach need to be evaluated.

**Materials and methods:**

A retrospective study was performed on patients admitted to our hospital who were diagnosed with unilateral or bilateral moyamoya disease (MMD) associated with ruptured aneurysms in moyamoya vessels or on collaterals. These aneurysms were treated with PAO, and the clinical outcome was recorded.

**Results:**

Eleven patients were aged 54.7 ± 10.4 years, and six patients were male (54.5%, 6/11). The aneurysms in 11 patients were single and ruptured, and the average size was 2.7 ± 0.6 mm. Three (27.3%, 3/11) aneurysms were located at the distal anterior choroidal artery, 3 (27.3%, 3/11) were at the distal lenticulostriate artery, 3 (27.3%, 3/11) were at the P2–3 segment of the posterior cerebral artery, 1 (9.1%, 1/11) was at the P4–5 segment of the posterior cerebral artery, and 1 was at the transdural location of the middle meningeal artery. Among the 11 aneurysms, PAO by coiling was performed on 7 (63.6%, 7/11), and Onyx casting was performed on 4 (36.4%, 4/11). Of 11 patients, 2 (18.2%, 2/11) suffered intraoperative hemorrhagic complications. During follow-up, all patients had good outcomes with a modified Rankin scale score of 0–2.

**Conclusion:**

As a last resort, the application of PAO with coiling or casting Onyx for ruptured aneurysms in moyamoya vessels or on collaterals may be safe with an acceptable clinical outcome. However, patients with MMD may not always achieve expected health outcomes, and PAO for the aneurysm can bring only temporary relief.

## 1. Introduction

Moyamoya disease (MMD) is a progressive stenosis or occlusion of the intracranial internal carotid artery (ICA) with the formation of moyamoya vessels and collateral vasculature to preserve cerebral perfusion (1, 2). In China, MMD is not uncommon, and the incidence and prevalence in mainland China are 0.59 and 1.01 per 100,000 person-years, respectively (3). The rates of MMD in China were greater than those in the United States and Europe but lower than those from other East Asian countries, such as Japan and South Korea (3).

In MMD, intracranial residual arteries had alterations in cerebral blood flow, and these arteries burdened more blood flow stress than that of the normal state; therefore, aneurysms can occur, with a prevalence from 3 to 14% (4, 5). These aneurysms can mainly be located on the unaffected major trunk artery, in moyamoya vessels or on collaterals (6, 7). Aggressive treatment for associated aneurysms in MMD should be provided, especially for ruptured aneurysms, to avoid rebleeding (8). Aneurysms in moyamoya vessels or on collaterals are difficult to treat through craniotomy because it is difficult to localize and identify them (9–11).

Currently, endovascular treatment (EVT) is an option for associated aneurysms in MMD that can overcome the drawback of craniotomy (7). However, the parent artery of this type of aneurysm is often thin and tortuous, and selective catheterization can be difficult (12). In addition, during EVT, preservation of the parent artery is also difficult (13). Therefore, parent artery occlusion (PAO) may often be the last resort. However, is PAO safe for these patients? To address this question, we performed a retrospective study.

## 2. Materials and methods

A retrospective study was performed for patients with MMD associated with ruptured aneurysms who underwent PAO from January 2015 to September 2022. This study was approved by the Ethics Committee of the First Hospital of Jilin University (No. 2022-KS-110). All methods were performed in accordance with the relevant guidelines and regulations.

### 2.1. Inclusion and exclusion criteria

The inclusion criteria were as follows: (1) patients had ipsilateral or bilateral MMD associated with ruptured aneurysms, presenting with various intracranial hemorrhages. (2) Aneurysms were located in moyamoya vessels or on collaterals, including the distal anterior choroidal artery (AchA), lenticulostriate artery (LSA), distal posterior cerebral artery (PCA) and middle meningeal artery (MMA). (3) There was a path to catheterize the aneurysm to perform PAO.

Those aneurysms on the major trunk of the intracranial arteries, in which the parent artery can be preserved during EVT, were excluded. Those aneurysms in which the parent artery was too thin, that could not be distinguished or in which EVT failed or abandoned were excluded. However, the clinical data of these patients were still summarized to provide more information about associated aneurysms in MMD.

### 2.2. Perioperative data collection

Perioperative data collected included the sex/age of the patient, Hunt-Hess grading, variations in intracranial hemorrhage, side of MMD, Suzuki staging of MMD, size of the aneurysm, and location of the aneurysm.

### 2.3. EVT scheme and strategy

All patients were treated under general anesthesia *via* a transfemoral approach. Digital subtraction angiography (DSA) with three-dimensional reconstruction confirmed the angioarchitecture of the aneurysm. After the best projection degree showed both the aneurysm neck and the parent artery, under roadmap guidance, the microcatheter was used to catheterize the parent artery and the aneurysm.

If the parent artery was not tortuous or thick enough and if the aneurysm was not far from the major trunk artery, an Echelon-10 microcatheter (Medtronic, Irvine, California, USA) under 0.014-inch microguidewire navigation was used to perform PAO with coiling. If the Echelon-10 microcatheter was too thick to pass through the parent artery or if the aneurysm was too far from the major trunk artery and periphery, a soft Marathon microcatheter (Medtronic, Irvine, California, USA) with a 1.5F tip under 0.012- or 0.010-inch microguidewire navigation was used to perform PAO with casing Onyx (Medtronic, Irvine, California, USA) (14, 15).

After the microcatheter was positioned in the proximal segment of the parent artery or in the aneurysm, super selective angiography was performed to confirm the position of the microcatheter tip. Then, PAO can be performed by coiling both the parent artery and the aneurysm, coiling only the proximal segment of the parent artery, or occluding both the parent artery and the aneurysm with Onyx casting. During EVT, if urgent intraoperative bleeding occurred, continuous coiling or Onyx casting was used to stop the bleeding.

### 2.4. EVT outcome and prognostic evaluation

PAO methods, EVT complications and new neurological deficits were recorded. Follow-up angiographic outcomes, including using DSA, magnetic resonance angiography (MRA) or computed tomography angiography, were recorded. During the follow-up by telephone interview, the modified Rankin scale (mRS) was used for clinical outcome assessment. A good outcome was defined as an mRS score of 0–2 (16).

## 3. Results

### 3.1. General information

Eleven patients who met the inclusion criteria were enrolled, aged 39–69 years (mean 54.7 ± 10.4 years), and six patients were male (54.5%, 6/11). All patients had intracranial hemorrhages, including 3 (27.3%, 3/11) patients with subarachnoid hemorrhage (SAH), 3 (27.3%, 3/11) patients with intracerebral hematoma (IH), 3 (27.3%, 3/11) patients with intraventricular hemorrhage (IVH), 1 (9.1%, 1/11) patient with SAH and IVH, and 1 (9.1%, 1/11) patient with IH and IVH. The HH grade was I in 2 (18.2%, 2/11) patients, II in 4 (36.4%, 4/11) patients, and III in 5 (45.5%, 5/11) patients.

### 3.2. Imaging characteristics

In 11 patients, 6 (54.5%, 6/11) had unilateral MMD, and 5 (45.5%, 5/11) had bilateral MMD. The Suzuki staging grade was II in 1 (9.1%, 1/11) patient, III in 3 (27.3%, 3/11) patients, IV in 5 (45.5%, 5/11) patients, and V in 2 (18.2%, 2/11) patients. Regarding the location of the aneurysms, 3 (27.3%, 3/11) aneurysms were located at the distal AchA, 3 (27.3%, 3/11) were at the distal LSA, 3 (27.3%, 3/11) were at the P2–3 segment of the PCA, 1 (9.1%, 1/11) was at the P4–5 segment of the PCA, and 1 was at the transdural location of the MMA. All patients had single aneurysms, and the size of the aneurysms ranged from 2 to 4 mm (mean 2.7 ± 0.6 mm).

### 3.3. PAO method

Among 11 aneurysms, 4 (36.4%, 4/11) were treated *via* PAO by coiling the parent artery, and 3 (27.3%, 3/11) were treated *via* PAO by coiling both the parent artery and the aneurysm. In these 7 cases, the parent arteries were thick enough to pass the Echelon-10 microcatheter to perform coiling. Of the other 4 aneurysms, 2 (18.2%, 2/11) were treated *via* PAO by occluding both the parent artery and the aneurysm by casting Onyx, and 2 (18.2%, 2/11) were treated *via* PAO by occluding the parent artery by casting Onyx. In these 4 cases, the parent arteries were thin, and the Marathon microcatheter was chosen to perform Onyx casting.

**Figure 1:**
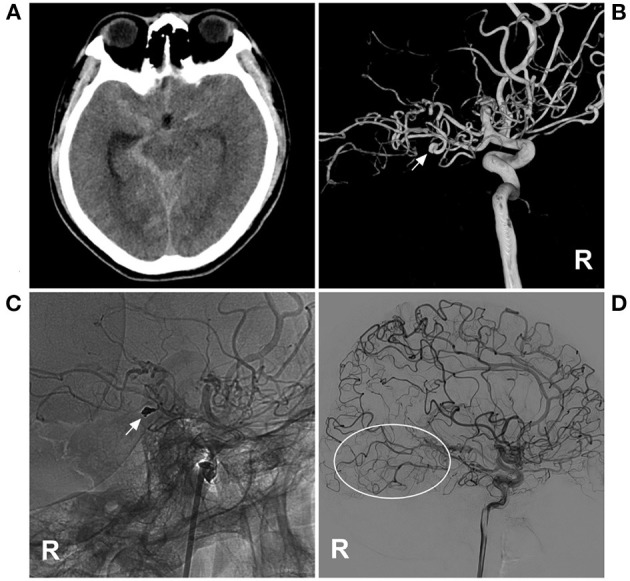
PAO of the PCA aneurysm in MMD in case 4 in Table 1. **(A)** CT showed subarachnoid hemorrhage focusing on the right ambient cistern. **(B)** Three-dimensional DSA of the right ICA showed an aneurysm (arrow) at the P2–3 segment of the PCA. **(C)** Unsubtracted DSA of the right ICA showed that the aneurysm and proximal parent artery underwent PAO by coiling (arrow). **(D)** Follow-up DSA of the right ICA showed no recurrence of the aneurysm, and the collateral circulation beyond the coils was abundant (circle). CT, computed tomography; DSA, digital subtracted angiography; ICA, internal carotid artery; MMD, moyamoya disease; PAO, parent artery occlusion; PCA, posterior cerebral artery; R, right.

**Figure 2:**
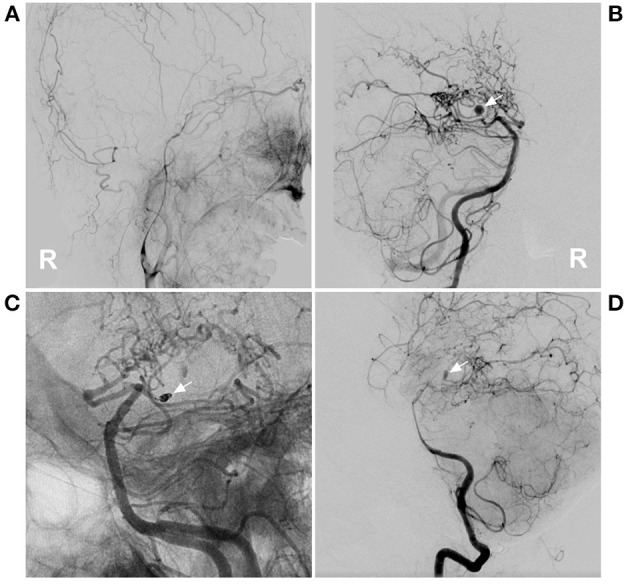
PAO of the PCA aneurysm in case 5 in Table 1. **(A)** DSA of the right carotid artery showed that the extracranial ICA was thin, and the intracranial ICA was MMD. **(B)** DSA of the right vertebral artery showed an aneurysm located on the P2–3 segment of the right PCA (arrow). **(C)** Unsubtracted DSA showed PAO of the P2–3 segment of the PCA by coiling (arrow). **(D)** DSA showed that the aneurysm (arrow) could be seen by reflux from the collateral circulation in the late artery phase of the arterial angiogram. DSA, digital subtracted angiography; ICA, internal carotid artery; MMD, moyamoya disease; PAO, parent artery occlusion; PCA, posterior cerebral artery; R, right.

**Figure 3:**
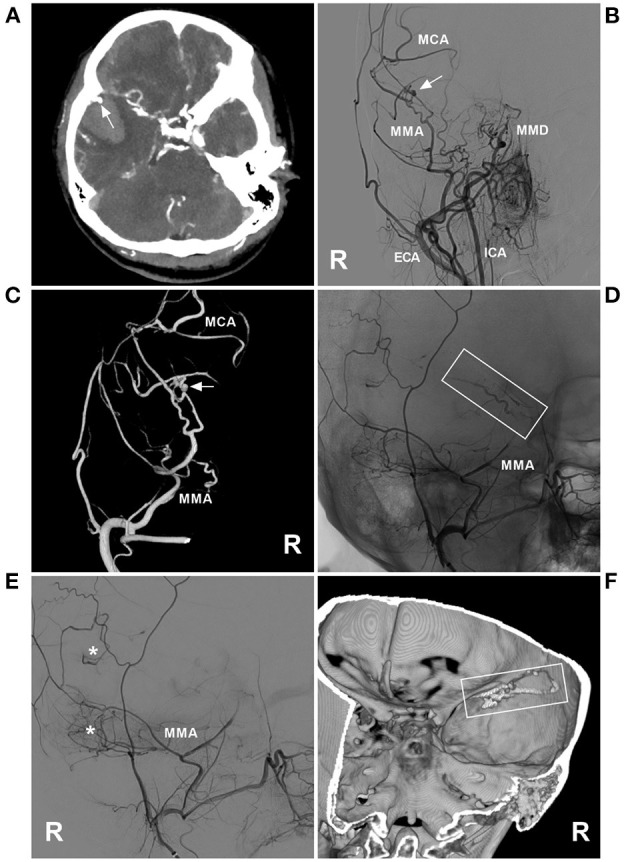
PAO of the MMA aneurysm in case 11 in Table 1. **(A)** Enhanced CT showing hemorrhage of the temporal lobe. An aneurysm (arrow) is indicated at the rim of the hematoma. **(B)** DSA of the right carotid artery showing MMD in the ICA; an aneurysm (arrow) was at the MMA. **(C)** Three-dimensional DSA of the right ECA showing an aneurysm (arrow) at the transdural location of the MMA; then, the MCA was continued. **(D)** Unsubtracted DSA showing the MMA as the parent artery to the aneurysm was occluded with Onyx (frame). **(E)** DSA of the right ECA showing that the petrous branch of MMD had anastomoses (asterisks) with the MCA. **(F)** Xper-CT showing the location of Onyx casting (frame). CT, computed tomography; DSA, digital subtracted angiography; ECA, external carotid artery; ICA, internal carotid artery; MCA, middle cerebral artery; MMA, middle meningeal artery; MMD, moyamoya disease; R, right.

**Figure 4:**
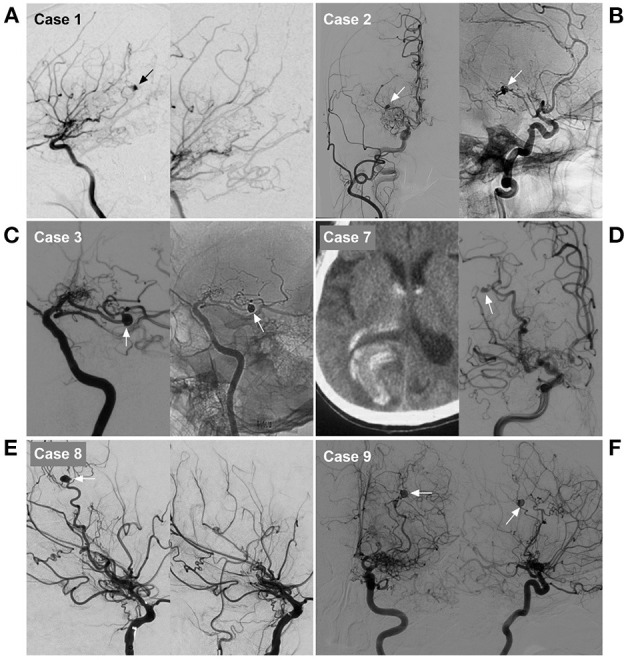
Imaging characteristics of cases 1, 2, 3, 7, 8 and 9 in Table 1. **(A)** Case 1: Left, DSA of the ICA showed an aneurysm on the distal AchA (arrow). Right: Postoperative DSA showing that both the parent artery and aneurysm were occluded with Onyx. **(B)** Case 2: Left, DSA of the carotid artery showed an aneurysm on the distal AchA (arrow). Right: Postoperative DSA showing that both the parent artery and aneurysm were occluded with coiling (arrow). **(C)** Case 3: Left, DSA of the ICA showed an aneurysm on the distal AchA (arrow). Right: Postoperative DSA showing that both the parent artery and aneurysm were occluded with coiling (arrow). **(D)** Case 7: Left, CT showed hemorrhage of the right occipital lobe into the lateral ventricle. Right: DSA of the ICA showing the aneurysm (arrow) located on the P4–5 segment of the PCA. **(E)** Case 8: Left, DSA of the ICA showed an aneurysm on the distal LSA (arrow). Right: Postoperative DSA showing that both the parent artery and aneurysm were occluded. **(F)** Case 9: DSA of the ICA in the anterior-posterior view (left) and lateral view (right) showed an aneurysm on the distal LSA (arrow). AchA, anterior choroidal artery; CT, computed tomography; DSA, digital subtracted angiography; ICA, internal carotid artery; LSA, lenticulostriate artery; PAO, parent artery occlusion; PCA, posterior cerebral artery.

After EVT, of 11 aneurysms, 10 aneurysms could not be seen on immediate postoperative angiography (Figure 1). One aneurysm [case 5 (Table 1)] could be seen by reflux from the collateral circulation (Figure 2). The other EVT details are shown in Figures 3, 4.

**Table 1:** Clinical data in cases treated for PAO.

**No**.	**Age/sex**	**Onset**	**HH grading**	**Side of MMD**	**MMD grading**	**Aneurysm location**	**Aneurysm size**	**PAO methods**	**Complication**	**Imaging follow-up**	**Telephone follow-up**	**mRS score**
1	39/F	IVH	III	Bilateral	IV	Distal AchA	3 mm	Occluding both the parent artery and aneurysm with Onyx	No	DSA, no recurrence	28 months	2
2	66/F	SAH + IVH	II	Bilateral	IV	Distal AchA	2.5 mm	Coiling both the parent artery and aneurysm	Intraoperative bleeding, no postoperative new deficit	None	8 months	1
3	60/M	IH	III	Bilateral	IV	Distal AchA	4 mm	Coiling both the parent artery and aneurysm	No	DSA, no recurrence	48 months	2
4	46/F	SAH	II	Unilateral	III	P2–3	2.5 mm	Coiling both the parent artery and aneurysm	No	DSA, no recurrence	8 months	0
5	57/M	SAH	I	Unilateral	V	P2–3	3 mm	Coiling the parent artery	No	None	6 months	0
6	57/M	SAH	II	Bilateral	V	P2–3	3 mm	Coiling the parent artery	No	DSA, no recurrence	18 months	1
7	69/M	IH + IVH	III	Unilateral	III	P4–5	2 mm	Occluding both the parent artery and aneurysm with Onyx	No	None	48 months	2
8	56/M	IVH	I	Unilateral	III	Distal lenticulostriate artery	3 mm	Coiling the parent artery	Intraoperative bleeding, postoperative hemiparesis	MRA, no recurrence	25 months	1
9	40/F	IVH	II	Bilateral	II	Distal lenticulostriate artery	2 mm	Occluding the parent artery with Onyx	No	None	48 months	1
10	65/F	IH	III	Unilateral	IV	Distal lenticulostriate artery	2 mm	Coiling the parent artery	No	None	78 months	2
11	47/F	IH	III	Unilateral	IV	MMA at transdural location	2.5 mm	Occluding the parent artery with Onyx	No	None	1 month	1

AchA, anterior choroidal artery; DSA, digital subtracted angiography; F, female; HH, hunt-hess grading; IH, intracerebral hemorrhage; IVH, intraventricular hemorrhage; M, male; MMA, middle meningeal artery; MMD, moyamoya disease; MRA, magnetic resonance angiography; mRS, modified Rankin scale; PAO, parent artery occlusion; P2–3, second and third segment of posterior cerebral artery; P4–5, fourth and fifth segments of the posterior cerebral artery; SAH, subarachnoid hemorrhage.

### 3.4. EVT results

#### 3.4.1. Postoperative results and complications

Of the 11 patients, 2 (18.2%, 2/11) suffered intraoperative bleeding, and continuous EVT stopped the bleeding. After PAO, 10 (90.9%, 10/11) patients recovered well without new postoperative deficits, and 1 (9.1%, 1/11) patient who suffered a complication developed hemiparesis.

#### 3.4.2. Long-term follow-up

Among 11 patients, 5 (45.5%, 5/11) underwent follow-up imaging, including 4 DSA and 1 MRA, and had no recurrence of the aneurysms. During telephone interviews during the period of 1–78 months postoperatively (mean 28.7 ± 24.1 months), of 11 patients, 2 (18.2%, 2/11) had an mRS score of 0, 5 (45.5%, 5/11) had an mRS score of 1, and 4 (36.4%, 4/11) had an mRS score of 2. The clinical data are summarized in Table 1.

### 3.5. Clinical data with unsuccessful or abandoned EVT

In addition to the above 11 patients who met the inclusion criteria, we also included 5 patients with ruptured aneurysms in moyamoya vessels or on collaterals in the same period. These 5 patients had unsuccessful EVT or abandoned it altogether.

The five patients, including 2 females and 3 males were aged 36–70 years. They all suffered from intracranial hemorrhage. Of them, 4 had bilateral MMD, and 1 had unilateral MMD. The Suzuki staging grade was III in 2 patients and IV in 3 patients. One aneurysm was located at the distal AchA, and 4 were located in moyamoya vessels. The cause of unsuccessful or abandoned EVT was aneurysm regression in 1 patient, no path of EVT in 3 patients, and a poor state in 1 patient. The patient with a poor state had no chance of treatment and died. Two patients accepted conservative treatment, 1 patient suffered re-hemorrhage after a follow-up of 48 months, and 1 patient was still in a stable state. Two patients accepted surgical resection of the aneurysm and were in a stable state. These data are summarized in Table 2.

**Table 2:** Clinical data in cases with unsuccessful or abandoned EVT.

**No**.	**Age/sex**	**Onset**	**HH grading**	**Side of MMD**	**MMD grade**	**Aneurysm location**	**Aneurysm size**	**Cause of unsuccessful lor abandoned EVT**	**Sequent management**	**Follow-up**	**mRS score**
1	70/M	IVH	I	Unilateral	III	Distal AchA	2 mm	Intraoperative angiography showing aneurysm regression	Conversative treatment	21 months	0
2	36/F	IH	II	Bilateral	III	Moyamoya vessel	5 mm	No path of EVT	Surgical resection	47 months	0
3	53/F	IH	III	Bilateral	IV	Moyamoya vessel	2 mm	No path of EVT	Surgical resection	36 months	3
4	50/M	IH	III	Bilateral	IV	Moyamoya vessel	1.5 mm	No path of EVT	Conversative treatment	48 months later, re-hemorrhage	3
5	53/M	IH	IV	Bilateral	IV	Moyamoya vessel	3 mm	Bad state of patient	No	No	5

AchA, anterior choroidal artery; F, female; HH, hunt-hess grading; IH, intracerebral hemorrhage; IVH, intraventricular hemorrhage; M, male; MMD, moyamoya disease; mRS, modified Rankin scale.

## 4. Discussion

In the development of MMD, brain blood flow is redistributed, and alterations in cerebral blood flow may result in the formation of moyamoya vessels and the dilation of non-occluded vessels as collateral passages (2, 5, 7, 17–20). Moyamoya vessels mainly refer to the proliferative LSA in the basal ganglia (13, 15). The collateral passage is defined as a blood vessel capable of compensating for the progressive occlusion of anterior circulation, including the posterior communicating artery and PCA, the AchA, the thalamoperforating artery, the posterior choroidal artery, and the ophthalmic artery (21).

Due to brain hemodynamic alterations and the fragile nature of moyamoya vessels and collaterals, aneurysms can develop in or on them (6, 13, 15, 20). In Larson et al.'s (22) systematic review about the location of the associated aneurysm in MMD, 33.7% of aneurysms were located at the non-major artery type, belonging to the type on moyamoya vessels or on collaterals. These aneurysms share the same clinical characteristics. Therefore, in our study, they are discussed together, including the total of 11 aneurysms that were on the distal AchA, LSA, distal PCA, and MMA (Table 1). In addition, data from five cases with unsuccessful or abandoned EVT were collected to supplement our study (Table 2).

**Figure 5:**
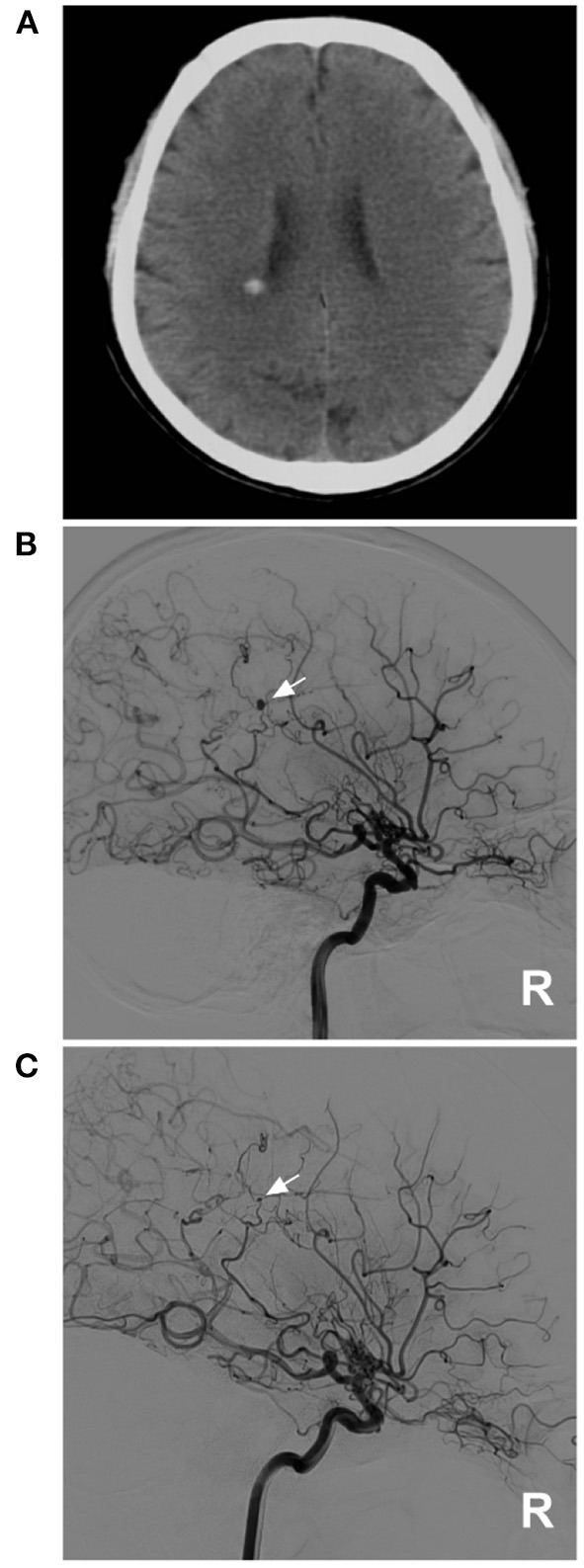
A diminishing aneurysm in case 1 in Table 2. **(A)** CT showed hemorrhage on the lateral ventricle wall. **(B)** DSA of the right ICA showed an aneurysm on the distal AchA (arrow), and MMD can be seen on this side. **(C)** One month later, repeat DSA of the right ICA showed that the aneurysm was diminishing, and only a remnant could be seen (arrow). AchA, anterior choroidal artery; CT, computed tomography; DSA, digital subtracted angiography; ICA, internal carotid artery; MMD, moyamoya disease; R, right.

**Figure 6:**
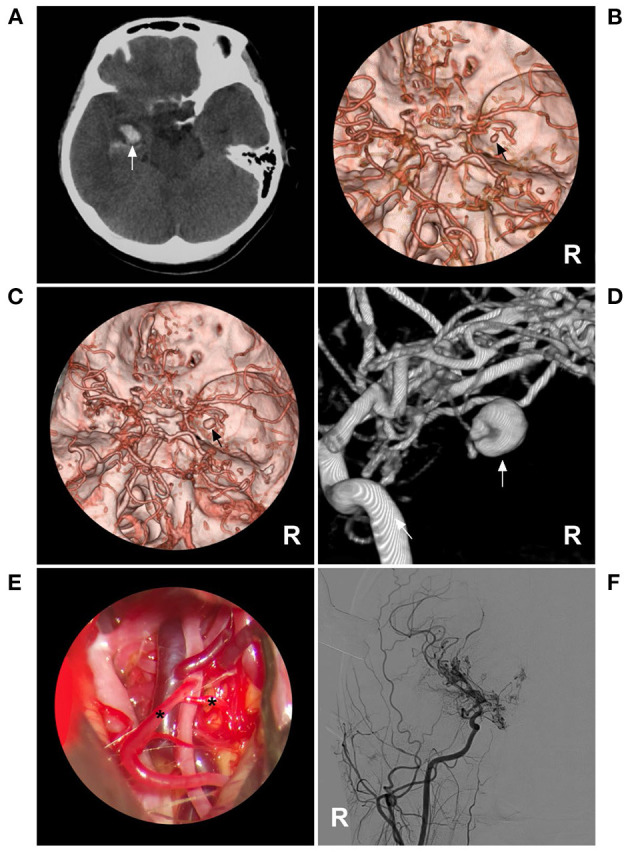
A growing aneurysm in case 2 in Table 2. **(A)** CT showed hemorrhage (arrow) in the right temporal lobe involving the ventricle. **(B)** CTA showed an aneurysm (arrow) in the right moyamoya vessels, and bilateral MMD can be seen. **(C)** One week later, repeated CTA showed that the aneurysm had grown (arrow). **(D)** Three-dimensional DSA of the right ICA showed that the aneurysm (arrow) vessels affected by MMD were enlarged. **(E)** Intraoperative image showing the vessels affected by MMD (asterisks); these vessels appeared thin and frail. **(F)** Postoperative DSA after aneurysm resection showed that the aneurysm had disappeared. CT, computed tomography; CTA, computed tomography angiography; DSA, digital subtracted angiography; ICA, internal carotid artery; MMD, moyamoya disease; R, right.

Due to the small caliber of moyamoya vessels and their passage as collateral circulation in MMD, the aneurysms that occur on them are small, usually 2–3 mm in size (9). In our report, the size was 2.7 mm, which confirmed the above report. For these aneurysms in moyamoya vessels or on collaterals, the natural history has not been clearly revealed; they can grow or regress (9, 23–26). For instance, in case 1 in Table 2, 1 month after rupture, the aneurysm in the distal AchA nearly disappeared (Figure 5). However, in case 2 in Table 2, 1 week after rupture, the aneurysm in the moyamoya vessel grew (Figure 6).

**Figure 7:**
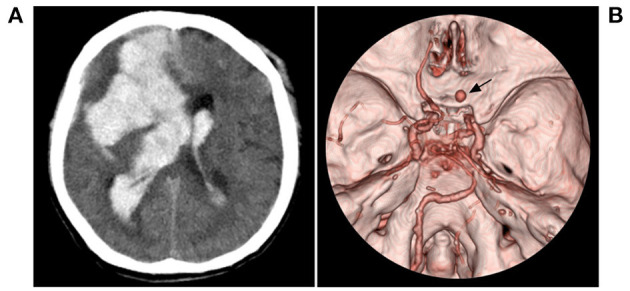
Fatal hemorrhage in case 5 in Table 2. **(A)** CT showed hemorrhage (arrow) in the right temporal lobe involving the ventricle. **(B)** CTA showed an aneurysm (arrow) in the right moyamoya vessels, and bilateral MMD can be seen. CT, computed tomography; CTA, computed tomography angiography; MMD, moyamoya disease.

Although some aneurysms in MMD may regress due to a decrease in blood flow or thrombosis, others are likely to present with rupture (23, 27–29). Aneurysm rupture may be fatal [Figure 7 (case 5 in Table 2)]. The rupture of aneurysms in moyamoya vessels or on collaterals suggests a poor natural history with an increased risk of hemorrhagic strokes (21, 26). In Kim et al. (7), during follow-up, 31.2% of aneurysms in the moyamoya vessel or on collaterals grew or ruptured, which showed that they had a poor natural history. In addition, these aneurysms are usually unstable pseudoaneurysms or dissections (26).

Therefore, aggressive treatment may be advocated (4, 30). Surgical challenges are attributable to the small size and deep location of these aneurysms, making intraoperative localization difficult (2). The fragile and delicate moyamoya vessels also add to the complexity of the procedure and increase the risk of damaging vital collaterals (31). Currently, EVT has emerged as an alternative to manage aneurysms in moyamoya vessels or on collaterals (6, 32). Ideally, during EVT, the patency of the parent artery should be preserved (14). In this study, 11 aneurysms in moyamoya vessels or on collaterals provided the opportunity for EVT; however, the parent arteries of these aneurysms were thin and tortuous, preservation of the parent artery was difficult, and PAO was the last resort (13). Due to the rarity of the procedure, the safety of PAO for these aneurysms is less known, so our study attempted to address this uncertainty.

When performing PAO, the aneurysm should be embolized to avoid rebleeding (Figure 1). However, due to the small caliber and tortuosity of the parent artery, sometimes PAO had to be targeted at the proximal segment of the parent artery away from the aneurysm. In our study, 6 patients had to only undergo PAO of the parent artery. When PAO was performed only in the proximal parent artery, sometimes, the aneurysm could still be seen in the late phase of arterial angiography *via* collateral circulation [case 5 (Table 1)] (Figure 2); however, PAO should be effective in preventing aneurysm rerupture because the stress of the reverse blood flow was low. At the 6-month follow-up, the patient was well.

Is PAO of the proximal segment of the parent artery safe to avoid symptomatic ischemia? In our report, we found that even away from the aneurysms, PAO of the proximal parent artery was also safe, and no severe new neurologic deficits occurred (33). This is because the sacrificed part of the parent artery to disrupt moyamoya collaterals was only a short segment of its conduit, not whole collateral channels, including the transdural path from the MMA (Figure 3) (10, 34). A nearby, rich collateral network could compensate for one conduit blockage. A previous report also showed the safety and effect of PAO; for instance, Kim et al. (10) reported successful PAO in 7 of 8 cases of MMD with ruptured aneurysms at collateral vessels, including the AchA and PCA, and no new neurological deficits were observed during the follow-up.

For PAO, the choice of embolic material had to be considered. Coiling is a good choice because coiling can preserve more parent arteries, but coiling requires thicker parent arteries, which are sometimes impossible to find due to the thin and tortuous parent arteries (35). In this study, among 11 aneurysms, PAO by coiling was performed in only 63.6% of cases. For other cases, a thin and soft microcatheter had to be chosen to cast the liquid embolic material, and even if away from the aneurysm, liquid embolic material can occlude the parent artery or penetrate the aneurysm by forward movement (36).

Currently, liquid embolic materials mainly include N-butyl-cyanoacrylate (NBCA) and Onyx (20). Onyx is a good embolic material that allows longer, slower, and more precise control of casting (15). In our report, we preferred Onyx due to its advantages. However, Onyx has the drawback that during casting, reverse dispersion can occlude more collateral circulation. To avoid reflux, the microcatheter should be in a wedge position; of note, the control of casing Onyx is relatively easy (10, 14).

Although PAO for ruptured aneurysms in moyamoya vessels or on collaterals was feasible in this study, ischemic and hemorrhagic complications had to be considered. Ischemic complications arose from the occlusion of the collateral circulation; in this study, no ischemic complications occurred. Hemorrhagic complications occurred in 2 cases due to fragile parent artery rupture. At the time, PAO had to be performed urgently to occlude the ruptured artery. Due to the low blood flow and thinness of the parent artery, it was not difficult to stop the bleeding.

Although our study with 11 cases showed PAO safety, we must be aware that the fate of the patients may not fundamentally change because in this study, most patients had late-Suzuki stage MMD (stages IV, V, and VI) (Tables 1, 2), the prognosis was worse, PAO for the aneurysm only can bring a moment of peace, and extracranial-intracranial bypass may be necessary to improve brain flow perfusion (37, 38).

## 5. Conclusions

Despite the potential risk of adjacent collateral vessel occlusion, in our study, the application of PAO for ruptured aneurysms in moyamoya vessels or on collaterals was feasible. The follow-up outcome was good. However, the fate of the patients may not achieve a fundamental change, and PAO for the aneurysm can only bring a moment of peace.

## 6. Limitations

Our study had limitations: the number of cases was small, the follow-up imaging was not sufficient, and only half of the patients had angiographic follow-up. This is because, in China, most patients refused follow-up radiology due to economic reasons. Therefore, the progress of MMD and its collaterals is not clear. However, in this study, the safety and effect of PAO can be suggested, which provides an alternative treatment for ruptured aneurysms in moyamoya vessels or on collaterals, which is important.

## Data availability statement

The original contributions presented in the study are included in the article/supplementary material, further inquiries can be directed to the corresponding author.

## Ethics statement

This study was approved by the Ethics Committee of the First Hospital of Jilin University (No. 2022-KS-110). All methods were performed in accordance with the relevant guidelines and regulations. The patients/participants provided their written informed consent to participate in this study. Written informed consent was obtained from the individual(s) for the publication of any potentially identifiable images or data included in this article.

## Author contributions

JY contributed to the conception. ZZ collected the data. JY and ZZ contributed to drafting the manuscript. ZZ and KX revised the manuscript. All authors read and approved the final manuscript.

## References

[1] Suzuki J Kodama N. Moyamoya disease: a review. Stroke. (1983) 14:104–9. 6823678 10.1161/01.str.14.1.104

[2] Lang M Moore NZ Witek AM Kshettry VR Bain MD. Microsurgical repair of ruptured aneurysms associated with moyamoya-pattern collateral vessels of the middle cerebral artery: a report of two cases. World Neurosurg. (2017) 105:1042.e5–10. 10.1016/j.wneu.2017.06.166 28698088

[3] Sun Y Zhou G Feng J Chen L Liu G Wang J. Incidence and prevalence of moyamoya disease in urban China: a nationwide retrospective cohort study. Stroke Vascul Neurol. (2021) 6:615–23. 10.1136/svn-2021-000909 33941642 PMC8717778

[4] Kim JH Kwon TH Kim JH Chong K Yoon W. Intracranial aneurysms in adult moyamoya disease. World Neurosurg. (2018) 109:e175–82. 10.1016/j.wneu.2017.09.127 28962951

[5] Lee JK Lee JH Kim SH Lee MC. Distal anterior choroidal artery aneurysm in a patient with moyamoya disease: case report. Neurosurgery. (2001) 48:222–5. 10.1227/00006123-200101000-00043 11152352

[6] Hou K Li G Luan T Xu K Yu J. The prospects and pitfalls in the endovascular treatment of moyamoya disease-associated intracranial aneurysms. Neurosurg Rev. (2021) 44:261–71. 10.1007/s10143-020-01261-y 32052219

[7] Kim S Jang CK Park EK Shim KW Kim DS Chung J. Clinical features and outcomes of intracranial aneurysm associated with moyamoya disease. J Clin Neurol. (2020) 16:624–32. 10.3988/jcn.2020.16.4.624 33029969 PMC7541995

[8] Zhang L Xu K Zhang Y Wang X Yu J. Treatment strategies for aneurysms associated with moyamoya disease. Int J Med Sci. (2015) 12:234–42. 10.7150/ijms.10837 25678840 PMC4323361

[9] Ho WM Görke AS Dazinger F Pfausler B Gizewski ER Petr O. Transcallosal, transchoroidal clipping of a hypothalamic collateral vessel aneurysm in Moyamoya disease. Acta Neurochir. (2020) 162:1861–5. 10.1007/s00701-020-04335-4 32306162 PMC7360665

[10] Kim SH Kwon OK Jung CK Kang HS Oh CW Han MH. Endovascular treatment of ruptured aneurysms or pseudoaneurysms on the collateral vessels in patients with moyamoya disease. Neurosurgery. (2009) 65:1000–4. 10.1227/01.NEU.0000345648.46096.CE 19834414

[11] Kanamori F Takasu S Ota S Seki Y. Prevention of the rerupture of collateral artery aneurysms on the ventricular wall by early surgical revascularization in moyamoya disease: report of two cases and review of the literature. World Neurosurg. (2018) 109:393–7. 10.1016/j.wneu.2017.10.059 29061453

[12] Leung GK Lee R Lui WM Hung KN. Thalamo-perforating artery aneurysm in moyamoya disease: case report. Br J Neurosurg. (2010) 24:479–81. 10.3109/02688697.2010.487128 20515265

[13] Ando M Maki Y Hojo M Hatano T. Ruptured saccular aneurysm of the lenticulostriate artery embolized without parent artery occlusion in a case of moyamoya disease. Neuroradiol J. (2022) 77:19714009221101307. 10.1177/19714009221101307 35545931 PMC9893168

[14] Chalouhi N Tjoumakaris S Gonzalez LF Dumont AS Shah Q Gordon D. Onyx embolization of a ruptured lenticulostriate artery aneurysm in a patient with moyamoya disease. World Neurosurg. (2013) 80:436.e7–10. 10.1016/j.wneu.2012.03.030 22484074

[15] Byeon Y Kim HB You SH Yang K. A ruptured lenticulostriate artery aneurysm in moyamoya disease treated with *Onyx embolization*. J Cerebrovasc Endovasc Neurosurg. (2022) 24:154–9. 10.7461/jcen.2021.E2021.06.011 34696549 PMC9260461

[16] Bacchus E Kate MP Benomar A Farzin B Raymond J Darsaut TE. Inter-rater reliability of the simplified Modified Rankin Scale as an outcome measure for treated cerebral aneurysm patients. Neurochirurgie. (2022) 68:488–92. 10.1016/j.neuchi.2022.04.003 35662528

[17] Hou K Li G Guo Y Xu B Xu K Yu J. Angiographic study of the transdural collaterals at the anterior cranial fossa in patients with moyamoya disease. Int J Med Sci. (2020) 17:1974–83. 10.7150/ijms.48308 32788876 PMC7415394

[18] Morioka M Hamada J Kawano T Todaka T Yano S Kai Y. Angiographic dilatation and branch extension of the anterior choroidal and posterior communicating arteries are predictors of hemorrhage in adult moyamoya patients. Stroke. (2003) 34:90–5. 10.1161/01.STR.0000047120.67507.0D 12511756

[19] Grabel JC Levine M Hollis P Ragland R. Moyamoya-like disease associated with a lenticulostriate region aneurysm: case report. J Neurosurg. (1989) 70:802–3. 2709122 10.3171/jns.1989.70.5.0802

[20] Hwang K Hwang G Kwon OK. Endovascular embolization of a ruptured distal lenticulostriate artery aneurysm in patients with moyamoya disease. J Korean Neurosurg Soc. (2014) 56:492–5. 10.3340/jkns.2014.56.6.492 25628809 PMC4303725

[21] Myeong HS Kim K Lee SH Yoo DH Cho YD Cho WS. Clinical significance of intracranial aneurysms in adult moyamoya disease. World Neurosurg. (2022) 164:e1034–e42. 10.1016/j.wneu.2022.05.092 35643409

[22] Larson AS Rinaldo L Brinjikji W Lanzino G. Location-based treatment of intracranial aneurysms in moyamoya disease: a systematic review and descriptive analysis. Neurosurg Rev. (2021) 44:1127–39. 10.1007/s10143-020-01307-1 32385590

[23] Chen H Hou K Wang X Xu K Yu J. Spontaneous recession of a posterior cerebral artery aneurysm concurrent with carotid rete mirabile and moyamoya-pattern collateral vessels: a case report. BMC Neurol. (2019) 19:51. 10.1186/s12883-019-1277-7 30940110 PMC6444516

[24] Rivera R Sordo J Badilla L Bravo E Riveros R Giacaman P. Middle cerebral artery occlusion with moyamoya-like vessels and aneurysms: a report of two cases. Interv Neuroradiol. (2014) 20:96–9. 10.15274/INR-2014-10014 24556306 PMC3971148

[25] Lee GY Cho BK Hwang SH Roh H Kim JH. Hydration-induced rapid growth and regression after indirect revascularization of an anterior choroidal artery aneurysm associated with moyamoya disease: a case report. J Cerebrovasc Endovasc Neurosurg. (2022) 2:1–6. 10.7461/jcen.2022.E2022.02.002 36153861 PMC10073769

[26] Yamada H Saga I Kojima A Horiguchi T. Short-term spontaneous resolution of ruptured peripheral aneurysm in moyamoya disease. World Neurosurg. (2019) 126:247–51. 10.1016/j.wneu.2019.02.193 30877003

[27] Kim YS Joo SP Lee GJ Park JY Kim SD Kim TS. Ruptured choroidal artery aneurysms in patients with moyamoya disease: two case series and review of the literatures. J Clin Neurosci. (2017) 44:236–9. 10.1016/j.jocn.2017.06.055 28694042

[28] Kawaguchi S Sakaki T Morimoto T Kakizaki T Kamada K. Characteristics of intracranial aneurysms associated with moyamoya disease: a review of 111 cases. Acta Neurochir. (1996) 138:1287–94. 8980731 10.1007/BF01411057

[29] Yang H Zhang L Wang M Wang J Chen L Lu H. Clinical features of and risk factors for intracranial aneurysms associated with moyamoya disease. Int J Stroke. (2020) 16:542–550. 10.1177/1747493020967224 33176625

[30] Gupta S Vicenty-Padilla J Lai PMR Zhou X Bernstock JD Chua M. Posterior cerebral artery aneurysm re-rupture following revascularization for moyamoya disease. J Stroke Cerebrovasc Dis. (2021) 30:106048. 10.1016/j.jstrokecerebrovasdis.2021.106048 34534774

[31] Ni W Jiang H Xu B Lei Y Yang H Su J. Treatment of aneurysms in patients with moyamoya disease: a 10-year single-center experience. J Neurosurg. (2018) 128:1813–22. 10.3171/2017.3.JNS162290 28841118

[32] Pathuri S Rodriguez P Mascitelli JR. Endovascular management of ruptured choroidal aneurysms associated with moyamoya syndrome: a two-patient case report and review of the literature. Clin Neurol Neurosurg. (2021) 209:106906. 10.1016/j.clineuro.2021.106906 34482116

[33] Ni W Xu F Xu B Liao Y Gu Y Song D. Disappearance of aneurysms associated with moyamoya disease after STA-MCA anastomosis with encephaloduro myosynangiosis. J Clin Neurosci. (2012) 19:485–7. 10.1016/j.jocn.2011.05.036 22281383

[34] Choulakian A Drazin D Alexander MJ. NBCA embolization of a ruptured intraventricular distal anterior choroidal artery aneurysm in a patient with moyamoya disease. J Neurointerv Surg. (2010) 2:368–70. 10.1136/jnis.2010.002253 21990650

[35] Schmalz PGR Alturki A Ogilvy CS Thomas AJ. Ruptured distal anterior choroidal artery aneurysm treated with super selective provocative testing and coil embolization. World Neurosurg. (2017) 105:1032.e19–22. 10.1016/j.wneu.2017.05.176 28599910

[36] Daou B Chalouhi N Tjoumakaris S Rosenwasser RH Jabbour P. Onyx embolization of a ruptured aneurysm in a patient with moyamoya disease. J Clin Neurosci. (2015) 22:1693–6. 10.1016/j.jocn.2015.05.017 26209917

[37] Ge P Ye X Zhang Q Zhang D Wang S Zhao J. Encephaloduroateriosynangiosis vs. conservative treatment for patients with moyamoya disease at late Suzuki stage. J Clin Neurosci. (2018) 50:277–80. 10.1016/j.jocn.2017.12.004 29366619

[38] Wang LX Wang H Hao FB Lv JH Zhang SH Han DS. Ivy Sign in moyamoya disease: a comparative study of the FLAIR vascular hyperintensity sign against contrast-enhanced MRI. AJNR Am J Neuroradiol. (2021) 42:694–700. 10.3174/ajnr.A7010 33664105 PMC8040985

